# B-cell repertoire dynamics after sequential hepatitis B vaccination and evidence for cross-reactive B-cell activation

**DOI:** 10.1186/s13073-016-0322-z

**Published:** 2016-06-16

**Authors:** Jacob D. Galson, Johannes Trück, Elizabeth A. Clutterbuck, Anna Fowler, Vincenzo Cerundolo, Andrew J. Pollard, Gerton Lunter, Dominic F. Kelly

**Affiliations:** Oxford Vaccine Group, Department of Paediatrics, University of Oxford and the NIHR Oxford Biomedical Research Center, Oxford, OX3 7LE UK; Paediatric Immunology, University Children’s Hospital Zürich, Zürich, 8032 Switzerland; Wellcome Trust Centre for Human Genetics, University of Oxford, Oxford, OX3 7BN UK; Medical Research Council Human Immunology Unit, Weatherall Institute of Molecular Medicine, Oxford, OX3 9DS UK

**Keywords:** Hepatitis B, B-cell repertoire, Vaccination, Polyreactive, VDJ, Antibody

## Abstract

**Background:**

A diverse B-cell repertoire is essential for recognition and response to infectious and vaccine antigens. High-throughput sequencing of B-cell receptor (BCR) genes can now be used to study the B-cell repertoire at great depth and may shed more light on B-cell responses than conventional immunological methods. Here, we use high-throughput BCR sequencing to provide novel insight into B-cell dynamics following a primary course of hepatitis B vaccination.

**Methods:**

Nine vaccine-naïve participants were administered three doses of hepatitis B vaccine (months 0, 1, and 2 or 7). High-throughput Illumina sequencing of the total BCR repertoire was combined with targeted sequencing of sorted vaccine antigen-enriched B cells to analyze the longitudinal response of both the total and vaccine-specific repertoire after each vaccine. ELISpot was used to determine vaccine-specific cell numbers following each vaccine.

**Results:**

Deconvoluting the vaccine-specific from total BCR repertoire showed that vaccine-specific sequence clusters comprised <0.1 % of total sequence clusters, and had certain stereotypic features. The vaccine-specific BCR sequence clusters were expanded after each of the three vaccine doses, despite no vaccine-specific B cells being detected by ELISpot after the first vaccine dose. These vaccine-specific BCR clusters detected after the first vaccine dose had distinct properties compared to those detected after subsequent doses; they were more mutated, present at low frequency even prior to vaccination, and appeared to be derived from more mature B cells.

**Conclusions:**

These results demonstrate the high-sensitivity of our vaccine-specific BCR analysis approach and suggest an alternative view of the B-cell response to novel antigens. In the response to the first vaccine dose, many vaccine-specific BCR clusters appeared to largely derive from previously activated cross-reactive B cells that have low affinity for the vaccine antigen, and subsequent doses were required to yield higher affinity B cells.

**Electronic supplementary material:**

The online version of this article (doi:10.1186/s13073-016-0322-z) contains supplementary material, which is available to authorized users.

## Background

B-cell repertoire diversity is a key feature of the humoral immune system, creating the potential for recognition of the wide variety of antigens likely to be encountered during an individual’s lifetime. The great diversity of this system, capable of generating up to 10^11^ unique B-cell receptor (BCR) variants [1], precludes analysis by conventional immunological techniques. Advances in next-generation sequencing technology now allow comprehensive characterization of B-cell samples at the level of their BCR DNA sequence. This technology is starting to yield insight into the dynamics of the B-cell response following antigen stimulation [2–7] and has great potential for increasing our understanding of humoral immunity and in vaccine development [8].

Laserson et al. showed that certain clones within the global B-cell repertoire undergo rapid expansions and contractions in response to vaccination [3]. However, these expansion dynamics were qualitatively different in different individuals and were not related to vaccine type or efficacy. We and others have also shown that the total repertoire undergoes stereotypic changes following vaccination—the repertoire has an increase in mutation and a decrease in diversity 7 days following vaccination, consistent with an increase in the number of mutated plasma cells (PCs) released into the peripheral blood at this time [2, 5, 6]. A small number of similar clones also appear to be produced in different individuals (the so-called public repertoire) after they receive the same antigen [2, 4, 7, 9]. Relating changes in the global repertoire to vaccine response is challenging as these repertoire dynamics may be confused with concurrent subclinical responses to irrelevant antigens [10]. Focusing on the public repertoire can overcome this to an extent [7], but the public repertoire is also enriched for clones specific to antigens that are commonly encountered by the population (e.g., tetanus toxoid, influenza) [10] and it is not clear to what extent the functional properties of the public antigen-specific repertoire and the private antigen-specific repertoire are different.

We designed the present study to overcome the difficulties of discerning the vaccine-specific from total repertoire and to give clearer insight into B-cell dynamics following vaccination. Hepatitis B (HepB) vaccine was used as the stimulus; this is a monovalent vaccine consisting of HepB surface antigen (HBsAg) and alum adjuvant. We hypothesized that the HepB vaccine response might be simpler to interpret than the response to a multivalent vaccine. In the United Kingdom, HepB vaccine is not given routinely so we were able to recruit HepB-naïve individuals and study the primary response. Primary HepB vaccination consists of three separate doses so we could also study how the response changed between the doses (Additional file 1: Figure S1). To this extent, we sequenced IgG transcripts from 500,000 total B cells on the day of and 7 days following each vaccine to gain insight into total repertoire dynamics. Cell sorting was also performed to enrich for vaccine-specific cells and sequencing these enabled generation of a vaccine-enriched sequence database. This database was combined with a HepB vaccine-enriched database generated in the same way from a previous HepB vaccine study [2], enabling us to distinguish the sequence clusters within the total repertoire that were vaccine-specific. Studying the vaccine-specific cluster dynamics gave a clearer signal of vaccine response and indicated a surprising role for previously generated memory B cells in the response to the first dose of vaccine.

## Methods

### Participants and vaccinations

Nine healthy subjects (aged 20–38 years) with no prior history of HepB vaccination or infection were recruited with informed consent in accordance with the Declaration of Helsinki and under approval from the Northampton Research Ethics Committee (13/EM/0036). As this was an observational study and given good response rates to this vaccine, nine participants was estimated to be a large enough sample to describe vaccine responses in this system. Participants were given a three-dose primary regime of monovalent HepB vaccine containing 10 μg HBsAg adsorbed on amorphous aluminum hydroxyphosphate sulfate (HBvaxPRO®, Sanofi Pasteur). Five participants were given a standard schedule (0, 1, and 7 months) and four participants were given an accelerated schedule (0, 1, and 2 months). Blood was taken immediately before vaccination, 7 days following each vaccination, and 1 month following the final vaccine (Additional file 1: Figure S1). Blood was transferred to a heparinized tube for processing within 4 h of collection.

### Anti-HBsAg antibody testing

Blood serum was isolated by centrifugation and tested for anti-HBsAg IgG antibody concentration by ELISA at the microbiology laboratory, John Radcliffe Hospital, Oxford.

### ELISpot

ELISpot was used to determine the number of HBsAg-specific memory and PCs using a previously described protocol [2]. Briefly, peripheral blood mononuclear cells (PBMCs) were first isolated from whole blood by density-gradient centrifugation. Multiscreen-IP 96-well ELISpot plates (Millipore) were coated with 2.5 μl/ml HBsAg (GSK). For detection of PCs, 200,000 PBMCs were added directly into each well of the plate, and for detection of memory cells, PBMCs were first incubated for 6 days in activation medium before addition to the plate. For each sample, the mean spot count was taken from six wells conducted in parallel.

### Cell sorting

B cells were magnetically enriched from PBMCs using CD19 microbeads (Miltenyi Biotec). For each sample, 500,000 B cells were isolated for sequencing the total repertoire and the remaining labeled with live/dead-aqua, CD19-FiTC, CD20-APCH7, CD27-PECy7, CD38-PE, HLA-DR-PerCPCy5, and HBsAg-APC. The specificity of HBsAg-APC staining was previously shown to be at least 50 % by use of a competition assay with unconjugated HBsAg [2]. On visits 2–7, viable, CD19^+^, CD20^+^, HBsAg^+^ B cells were isolated using a MoFlo cell sorter (Beckman Coulter) and on visits 2, 4 and 6, viable, CD19^+^, CD20^low/−^, CD27^+^, CD38^+^, HLA-DR^+^ PCs were isolated. Sorted cells were frozen at −80 °C in RLT buffer (Qiagen) until use.

### Repertoire sequencing

RNA was extracted from sorted cells using the RNeasy Mini Kit (Qiagen) and reverse transcription performed using SuperScript III (Invitrogen) and random hexamer primers (42 °C for 60 min, 95 °C for 10 min). PCR was conducted using the Multiplex PCR kit (Qiagen) with VH-family specific forward primers and IgG-specific reverse primers [11] (94 °C for 15 min, 30 cycles of 94 °C for 30 s, 58 °C for 90 s and 72 °C for 30 s, and 72 °C for 10 min). Amplicons were gel-extracted and purified prior to library preparation. Samples were multiplexed and sequenced across three 2 × 300 bp MiSeq (Illumina) runs.

### Sequence processing

Paired-end reads were joined using fastq-join (ea-utils) with default settings and filtered for minimum Phred quality of 30 over at least 75 % of bases. IMGT/HighV-Quest [12] was used for sequence annotation and unproductive sequences were removed. For total B-cell repertoire samples, subsampling to 75,000 sequences per sample was conducted and sequences were clustered into clonal lineages using a previously described algorithm [10]. To be considered part of the same cluster, sequences were required to have identical V and J genes and identical complementarity determining region (CDR) 3 length and were allowed 1 mismatch for every 15 nucleotides in the CDR3 sequence. The sequencing/PCR error rate of the methodology was previously estimated to be 0.0079 [10], so there will be some erroneous sequences in the dataset. The clustering approach used will group together reads arising from error as well as clonally related B cells. This means that, for analysis at the repertoire level, cluster size is used as a proxy for error-corrected B-cell abundance. For analysis of lineage trees of sequences within the clusters, we chose not to attempt removal of erroneous sequences due to difficulties in distinguishing error from somatic hypermutation, so it should be noted that these erroneous sequences will add some systematic noise to the analysis.

### Annotating clusters for putative antigenic specificity

Sequence data from sorted PC+ and HBsAg+ samples were compared with the total repertoire data to annotate clusters in the total repertoire for putative vaccine specificity. To match a sequence from the sorted data with a cluster in the total repertoire, it was required to utilize the same V and J gene segment and have a highly similar CDR3 amino acid sequence to the dominant sequence in the cluster (≥96 % identity). This matching approach means that sequences arising from sequencing/PCR error in the PC+ and HBsAg+ datasets will end up annotating the same cluster so do not need to be corrected for prior to matching. Sequences were also only matched back to participants from whom they were not obtained in order to reduce the effect of non-specific matching. PC+ and HBsAg+ samples from a previous study of HepB booster vaccination were also compared with the total repertoire data in the same way [2]. In addition, sequences from previously described HBsAg-specific clusters from the literature were compared with the total repertoire in the same way.

### Statistical analysis and graphing

Statistical analysis was performed using R [13], with ggplot2 [14] for graphing. Principal component analysis was performed using the prcomp function in the “stats” package [13]. Rarefaction analysis was conducted using the “iNEXT” package [15] using a q value of 0.

Lineage trees were constructed from sequence clusters using the “alakazam” package [16]. Lineages were only generated for clusters with at least 50 or 25 sequences depending on the analysis, and subsampling was performed if there were more than this number. The diversity of lineages was calculated using the Shannon entropy index with the “vegan” package [17], treating sets of unique sequences (nucleotide level) of the same length as independent species.

To determine antigen-driven selection pressures, the BASELINe framework was used with the focused test to analyze the distribution of mutations in the CDR and framework regions [18].

## Results

### Vaccine-specific cells are detected after vaccines 2 and 3 but not after vaccine 1

**Fig. 1:**
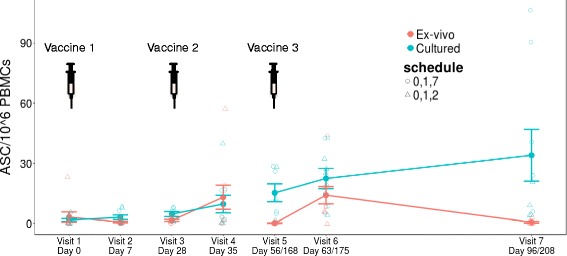
HBsAg-specific B-cell response kinetics determined by ELISpot and expressed as the number of antibody-secreting cells (*ASC*) per 10^6^ peripheral blood mononuclear cells (*PBMCs*) used in each assay. PBMCs are used ex vivo to detect PCs and are cultured to detect memory cells. Shown are the mean values ± standard error of the mean (SEM) for all nine participants. Detailed plots for each participant are shown in Additional file 1: Figure S2

ELISpot was used to determine the number of HBsAg-specific IgG PCs and memory cells in the peripheral blood at each visit (Fig. 1). The number of PCs detected 7 days following the first vaccine (visit 2) was negligible and PCs were only produced in detectable numbers 7 days following the second and third doses (visits 4 and 6, respectively). Despite considerable inter-individual variation in the response, with some participants having a greater PC peak at visit 4 and some at visit 6 (Additional file 1: Figure S2), the mean magnitude of the response was similar after each of these vaccines (at visit 4, mean 13 antibody-secreting cells (ASCs)/10^6^ PBMCs, range 0–58; at visit 6, mean 14 ASCs/10^6^ PBMCs, range 0–44). The number of memory cells detected increased steadily throughout the course of the study, reaching the greatest number at the final visit (Fig. 1). All participants had an anti-HBsAg antibody concentration greater than 10 mIU/ml at the end of the study. The response after the third vaccine dose was slightly greater in the group given the 0, 1, and 7 month schedule, although the study was not powered to detect such differences.

### Sequencing the total B-cell repertoire

Total IgG repertoire data were successfully obtained from all samples except one, where a blood sample could not be obtained. On average, 308,100 (range 240,000–355,900) raw illumina sequencing reads were obtained for each sample, of which 111,200 (range 75,870–136,100) remained after all filtering steps (Additional file 1: Table S1). Samples were subsampled to give 75,000 sequences for each and clustering performed. Clustering is used to group together clonally related sequences from each participant as well as for error correction by grouping together sequences which have arisen due to PCR or sequencing error. The threshold for clustering was set based on determining the CDR3 sequence nearest neighbor distribution for all sequences in the dataset (Additional file 1: Figure S3a). This distribution has two modes: the first represents sequences with clonal relatives (or erroneous sequences) and the second represents singletons. A clustering threshold of 1 nucleotide per 15 nucleotides was used to separate these two modes (Additional file 1: Figure S3a, b). On average, 12,212 (range 4081–19,304) clusters were generated from each sample. Rarefaction analysis indicated that our sampling of total clusters in the sample was tending towards saturation for most samples (Additional file 1: Figure S3c).

**Fig. 2:**
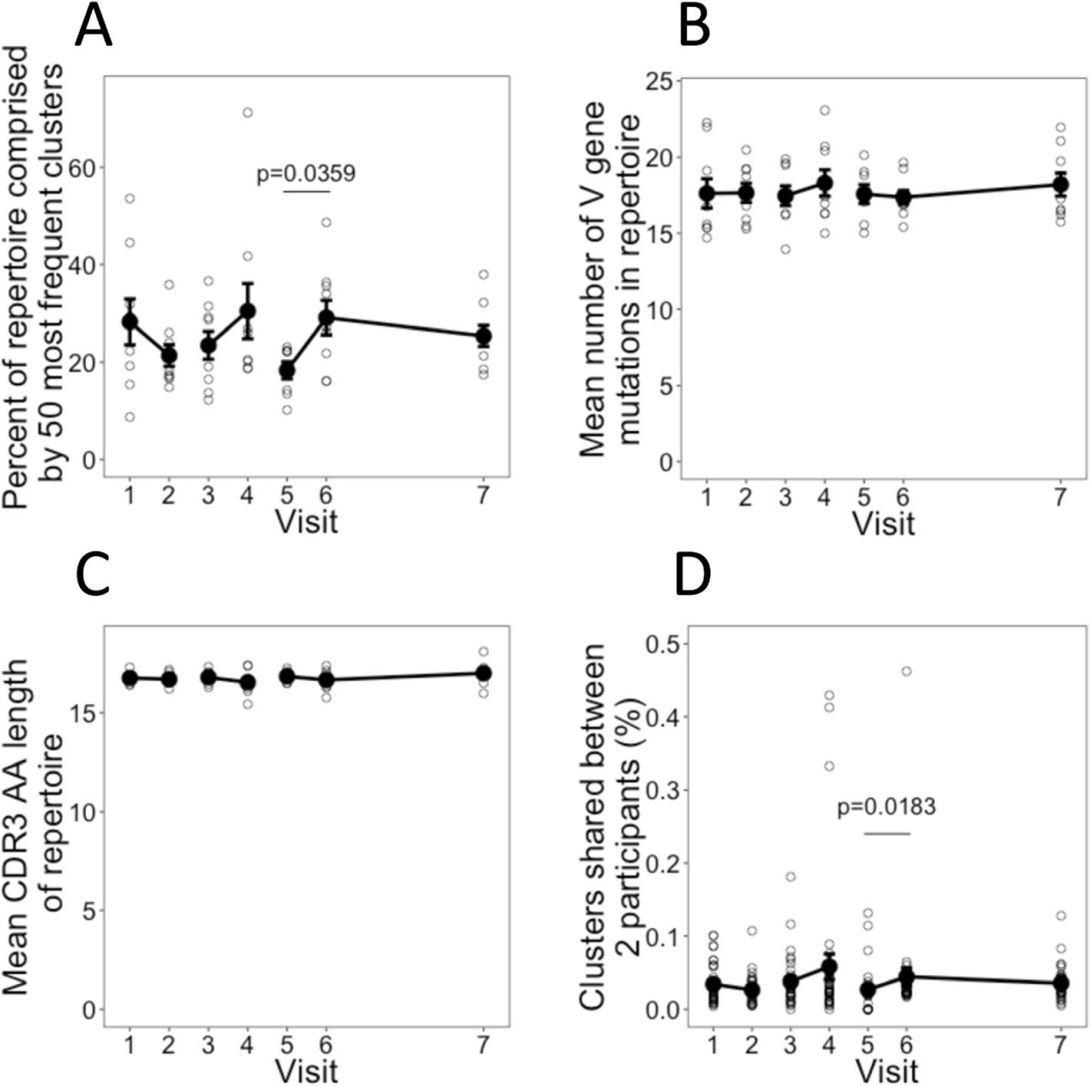
Fluctuations in the total repertoire. **a** Mean percentage of the total repertoire comprising the 50 most frequent clusters at each day (see also Additional file 1: Figure S3). **b** Mean number of V gene mutations from all sequences in the repertoire. **c** Mean CDR3 *AA* sequence length from all sequences in the repertoire. For **a**–**c**, error bars indicate ± SEM from nine participants. **d** The percentage of clusters shared by each pair of participants at each visit was determined (nine participants, giving 36 pairings). Shown are the mean values ± SEM of the percentage of clusters shared between each pair. Percent is calculated as (A∩B/size(A∪B)) × 100. Shared clusters are defined as those which share the same V and J gene segment and cluster center CDR3 nucleotide sequence. All *p* values were obtained from Mann–Whitney U tests

We previously showed an increase in cluster expansions, mutation, and sequence convergence between participants and a decrease in CDR3 amino acid (*AA*) sequence length following administration of HepB booster vaccine [2]; we thus determined the same properties in the current dataset. We did observe expansion of some clusters at each of the day 7 visits following each vaccine (Fig. 2a); however, this was only significant following the third dose of vaccine and there were also striking cluster expansions in two of the participants at baseline (Additional file 1: Figure S4). Changes in mutation and CDR3 length were not significant in this dataset (Fig. 2b, c). The size of the public repertoire did increase following the second and third vaccinations but the increase was only significant following the third vaccination (Fig. 2d).

### Enriching for the vaccine-specific repertoire

**Fig. 3:**
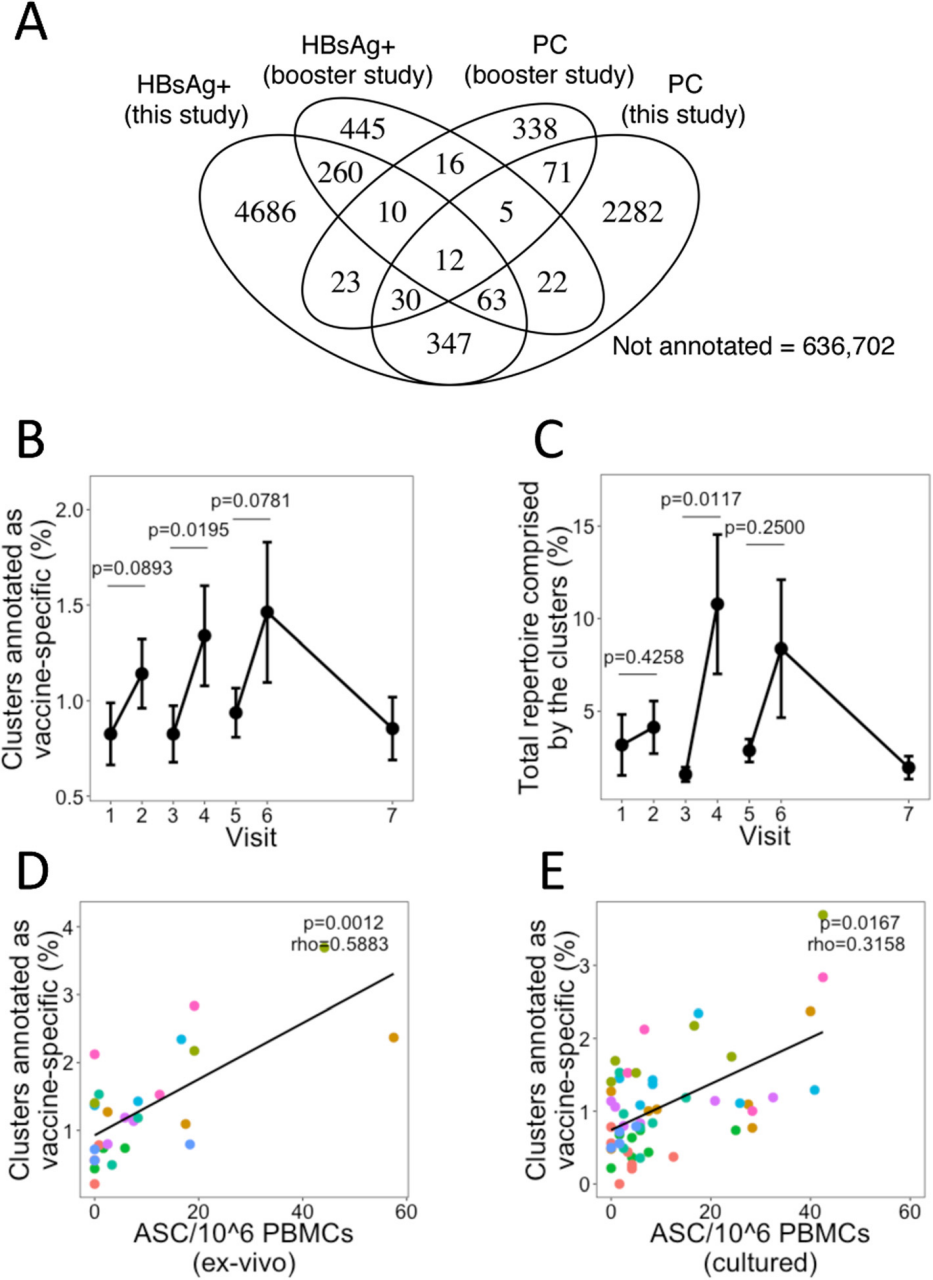
Enriching vaccine-specific clusters from the total repertoire. **a** The number of clusters from the total repertoire which were annotated by the four vaccine-enriched sequence datasets. **b** The percentage of abundant (>0.01 % of total repertoire) clusters at each visit that were characterized as vaccine-specific based on being annotated by at least two of the vaccine-enriched datasets. **c** Same as **b** but corrected for cluster size by considering the percentage of the repertoire comprising the vaccine-specific clusters. For **b** and **c**, mean values ± SEM are shown for all nine participants and *p* values were obtained from two-sided Mann–Whitney U tests. **d** Correlation (Spearman) between the percentage of abundant clusters characterized as vaccine-specific and PC numbers determined by ELISpot. *Different colored points* represent samples from the different participants. **e** Same as **d** but correlated with memory cell numbers determined by ELISpot. For **d** and **e**, samples where no cells were detected by ELISpot have been omitted

From each participant, we were able to isolate and sequence, on average, 26,720 HBsAg+ B cells in total across visits 2–7 and 2748 PCs on the day 7 visits (Additional file 1: Table S2). These data were combined with sequence data from 85,000 HBsAg+ cells and 3444 PCs obtained after HepB booster vaccine from our previous study [2] to create a large vaccine-enriched sequence database. Using these vaccine-enriched sequences to annotate the total repertoire data indicated that 5431 (0.85 %) of the total repertoire clusters had similarity (same V and J and ≥96 % CDR3 AA identity) to the HBsAg+ data obtained from this study, 2832 (0.44 %) had similarity to the PC data from this study, 833 (0.13 %) had similarity to the HBsAg+ data from the booster study, and 505 (0.08 %) had similarity to PC data from the booster study (Fig. 3a). The overlap in the clusters annotated by each of these datasets was significantly more than is expected by chance (Chi-square test, *p* < 0.0001 for each overlap).

As some of the sequences in these vaccine-enriched datasets may not actually be specific to the vaccine antigen, due to either non-specific staining for HBsAg+ or the inclusion of some non-specific PCs, we used a stricter definition of what we consider to be a vaccine-specific cluster in our total repertoire dataset. To be considered vaccine-specific, a cluster had to be annotated as similar to at least two of these datasets, and also be present at a frequency greater than 0.01 % (i.e., contain at least eight sequences). On average, 0.8 % of the frequent clusters were annotated as vaccine-specific at visit 1, but this number increased to 1.2 % by visit 2 (Fig. 3b). The number increased further 7 days after each of the two subsequent vaccines to peak at 1.5 % by visit 6. The clusters annotated as vaccine-specific tended to be large; when considering the percentage of the repertoire comprised by these clusters, these post-vaccination changes were thus more pronounced (Fig. 3c).

In total, 306 vaccine-enriched clusters (0.04 % of the total clusters and 0.53 % of frequent clusters identified) were found across all participants regardless of which visit they were detected on. The percentage of frequent clusters annotated as vaccine-specific in each participant at each visit correlated with the number of HBsAg-specific memory cells and PCs detected by ELISpot (Fig. 3d, e), giving validity to the technique for enriching vaccine-specific clusters.

### The vaccine-specific repertoire has distinct features when compared with the total repertoire

**Fig. 4:**
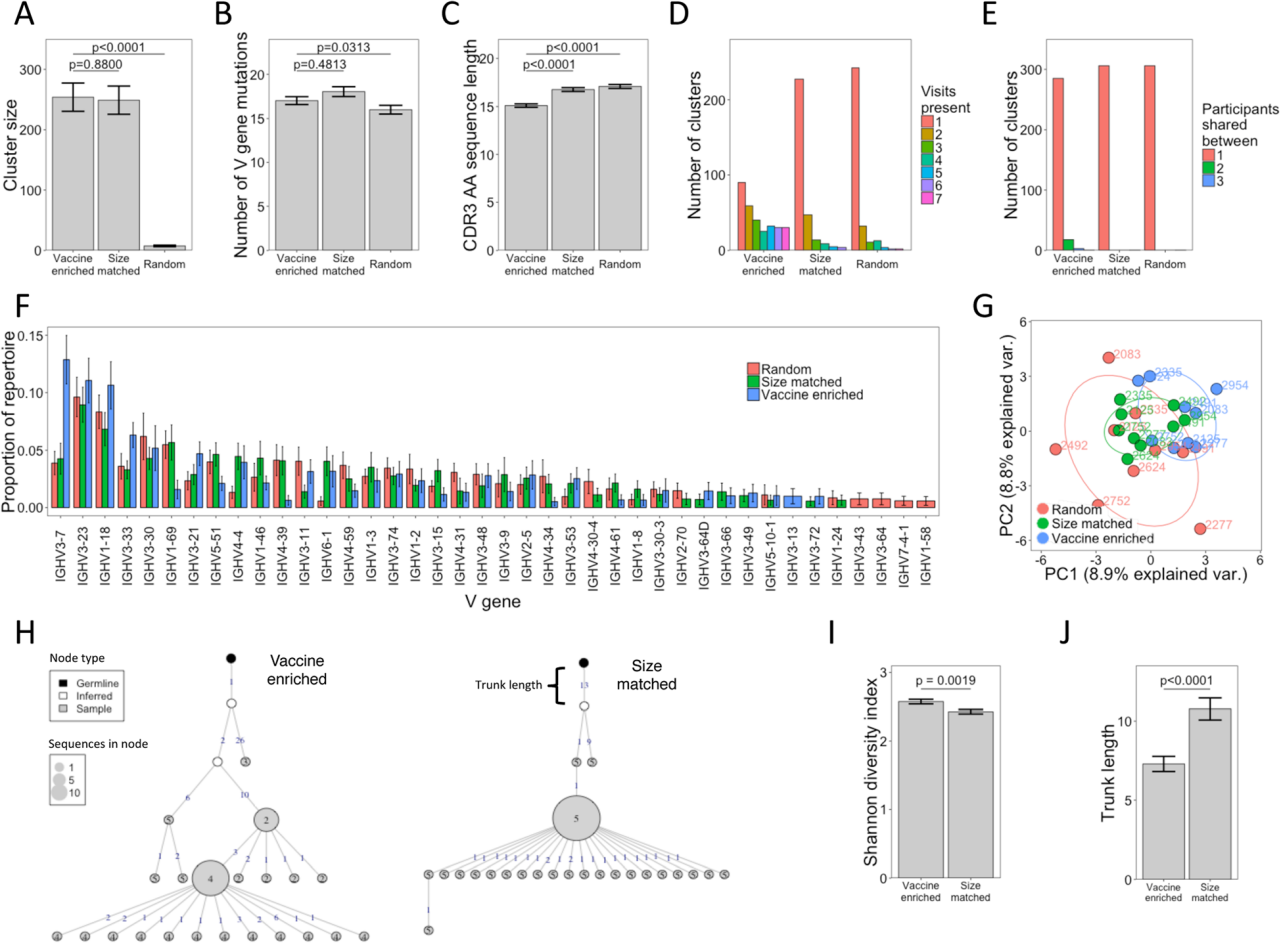
Comparing vaccine-specific clusters to a size-matched random set of clusters and a non-size-matched random set of clusters. **a**–**c** Differences in size, number of V gene mutations, and CDR3 *AA* sequence length of the clusters belonging to the three datasets. Shown are the mean values ± SEM of the 312 clusters in each dataset. Comparisons were performed using a *t*-test. **d** The number of visits where at least a single sequence from each cluster was found was determined and the number of clusters present at different numbers of visits in the different datasets plotted. **e** Same as **d** but counting the number of participants where a similar cluster was found (same CDR3 cluster center sequence and V/J gene usage). **f** The proportion of total clusters in the three different datasets utilizing different V gene segments in each participant. Error bars show mean values ± SEM of the nine participants. **g** Principal component analysis of V gene segment usage of the clusters in each of the datasets. **h** Representative lineage trees of a vaccine-specific and size-matched cluster. Each *node* represents a unique sequence within the cluster, with the size indicative of the number of duplicate sequences. The *number within the node* indicates the visit at which the sequence is first present. *Shading of the node* represents whether the sequence is found in the cluster, an inferred common ancestor to sequences found in the cluster, or the germline sequence. *Numbers on the edges of adjoining nodes* show the number of mutations between the sequences. **i**, **j** Lineage trees were created for all clusters which contained at least 50 sequences in the dataset (N = 200). Diversity was calculated for each cluster using the Shannon index and trunk length is the number of mutations between the most recent common ancestor and germline sequence. Shown are the mean values ± SEM. Comparisons were performed using a *t*-test

Next, we compared the properties of the vaccine-specific clusters and the total repertoire. For comparison, a random selection of 306 clusters that were not annotated as vaccine-specific was obtained from the total repertoire. As the size of the vaccine-specific clusters was much greater than that of the random clusters (Fig. 4a), a size-matched selection of random clusters was also obtained. The vaccine-specific clusters had similar levels of mutation to the random clusters but had significantly shorter CDR3 lengths (Fig. 4b, c). The vaccine-specific clusters were also more likely to be present both at multiple timepoints and in multiple individuals compared with the random clusters (Fig. 4d, e). It should be noted, however, that the vaccine-specific clusters are biased towards commonality between individuals due to the way that they were defined. The vaccine-specific clusters also had a distinct profile of V gene segment usage, with a striking increase in usage of IGHV3–7 (Fig. 4f, g).

To investigate the structure of the clusters, lineage trees were constructed from each of the vaccine-specific and size-matched clusters that contained at least 50 total sequences (Fig. 4h). Structure of the lineage trees can give insight into the relationships between clonal B cells within the cluster and pathways of BCR evolution that have occurred through proliferation and selection following antigen stimulation. Furthermore, by estimating the most recent common ancestor to the clone, it is possible to determine how mutated this sequence is compared with the germ line sequence (Fig. 4h; trunk length) and thus infer the maturation level of the initiating B cell for each clone [19]. Vaccine-specific lineages both contained a greater diversity of sequences (Fig. 4i) and had a shorter trunk length (Fig. 4j) than the size-matched random lineages, consistent with greater proliferation during the study period and more recent origin from germline.

### Dynamics of the vaccine-specific repertoire and evidence for IgG B cells at baseline participating in the response

**Fig. 5:**
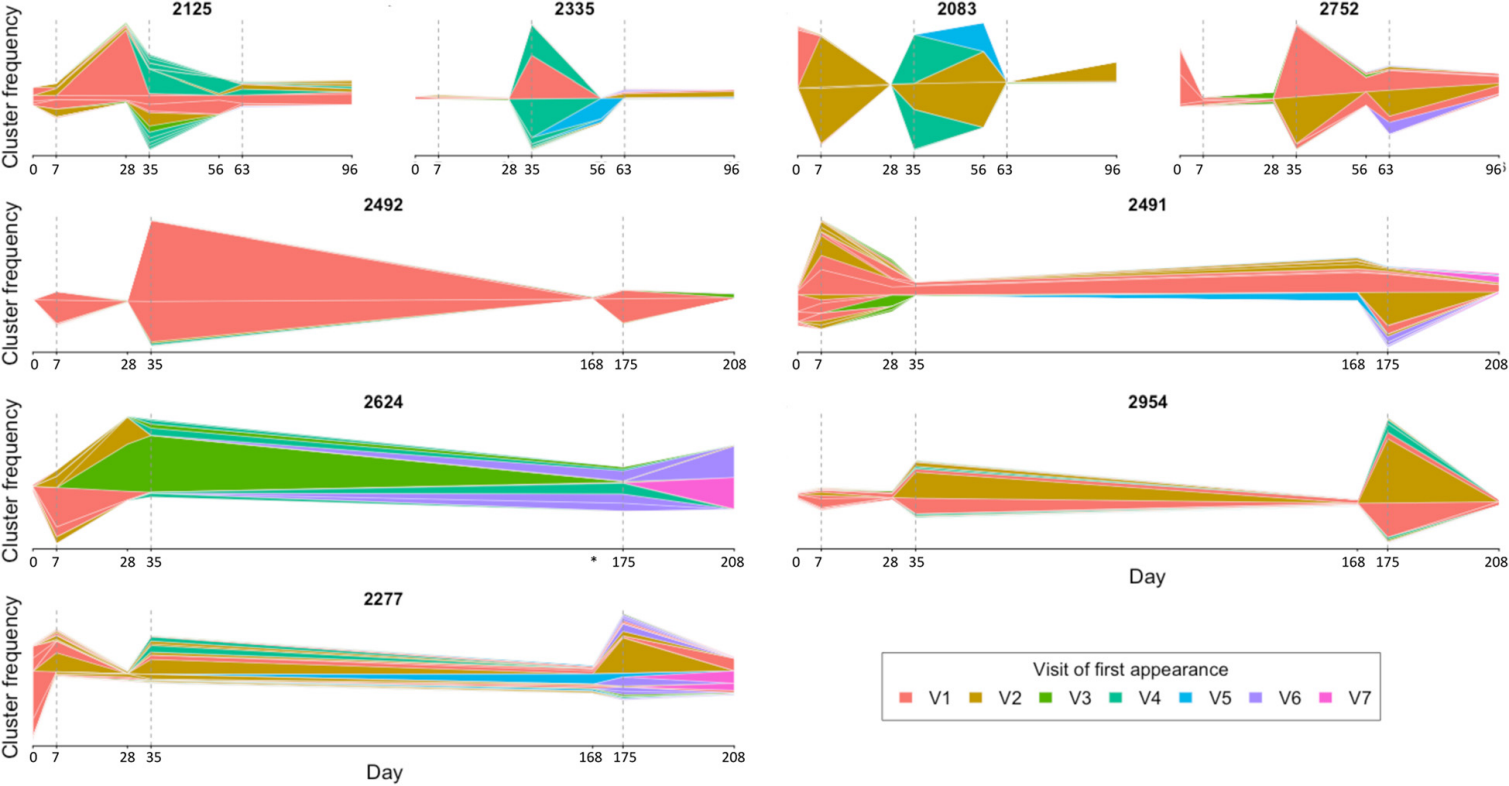
Kinetics of the vaccine-enriched clusters. The vaccine-enriched clusters were found in each participant and, at each day, the frequencies of these clusters are plotted as a stacked bar chart, centered to the middle of the *y-axis*. Clusters from each day are then joined using a horizontal stream to illustrate how the frequency of the clusters changes over time. The width of the stream represents the frequency of the cluster at that time and the color of the stream represents the first visit at which sequences from the cluster can be found. The *top four plots* are from participants who were given the accelerated vaccine schedule and the *bottom five plots* are from participants who were given the conventional vaccine schedule. *Dotted vertical lines* highlight the day 7 post-vaccination visits. *The day 168 blood sample was missing from this participant but the vaccine was still given on this day. See also Additional file 1: Figure S4 and Table S3 for kinetics of vaccine-enriched clusters as defined by similarity to previously publish HBsAg-specific monoclonal antibody sequences

**Fig. 6:**
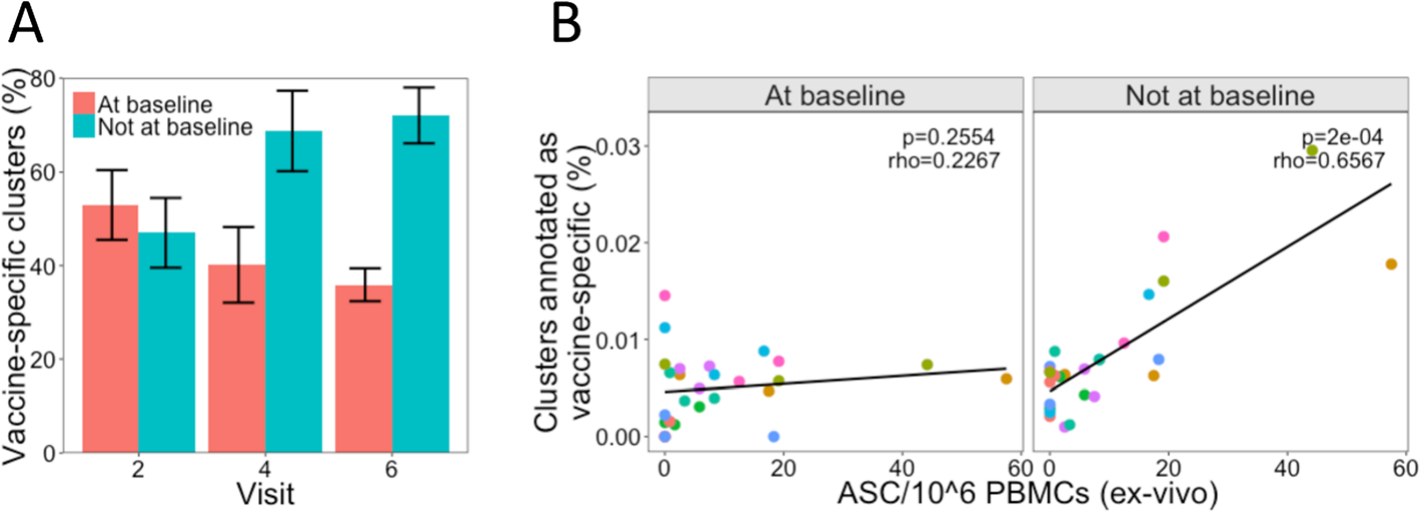
Vaccine-enriched clusters present at baseline. **a** In each of the nine participants, of the vaccine-specific clusters present at each post-vaccination time point (visit 2, 4, or 6), the percentage of these that were also present at baseline was determined. Mean ± SEM shown. **b** Correlation (Spearman) between the percentage of abundant clusters characterized as vaccine-specific and PC numbers determined by ELISpot, split according to whether the vaccine-specific clusters are also present at baseline. Different *colored points* represent samples from the different participants

Having identified a vaccine-specific repertoire with distinctive features, we next investigated the kinetics of these clusters in each participant over the course of the study (Fig. 5). There were considerable expansions of vaccine-specific clusters 7 days after each vaccine in the majority of cases and, within an individual, many of the same clusters were recurrently expanded after each vaccine dose. While the number of these expanded clusters was moderately correlated with the number of HBsAg-specific PCs detected by ELISpot in each participant (Fig. 3d), there were also striking expansions of the vaccine-specific clusters 7 days after the first vaccine despite no PCs being detected by ELISpot at this time. Furthermore, many of the clusters expanding after each vaccine were also present at low frequency at baseline despite none of the participants having previously encountered the vaccine antigen. The percentage of vaccine-specific clusters after each vaccine, which were also present at baseline, was similar after each vaccine dose (Fig. 6a). Correlating the percentage of frequent clusters annotated as vaccine-specific with the ELISpot data revealed a strong correlation when considering only the vaccine-specific clusters not present at baseline but no correlation when considering the vaccine-specific clusters that are present at baseline (Fig. 6b), suggesting that the baseline clusters are inherently different to those first identified at later visits and by ex vivo ELISpot.

One potential reason for finding these vaccine-specific clusters at baseline is due to insufficient stringency in our definition of what comprises a vaccine-specific cluster and that they are not actually specific to the vaccine. To investigate the presence at baseline of BCR sequences with specificity for the vaccine antigen, we collated a database of characterized HBsAg-specific antibodies from the literature. In total, 12 previously described sequences [20–23] mapped to clusters in our dataset based on CDR3 AA sequence identity, allowing one AA mismatch per 12 AAs (Additional file 1: Table S3). Although there were not enough of these clusters present to construct detailed plots of their kinetics, we did also observe that seven of these were found at baseline across six of the participants (Additional file 1: Figure S4).

We hypothesized that the clusters annotated as vaccine-specific that are also present at baseline were derived from memory B cells stimulated previously by a different antigen but that are also able to recognize HBsAg. These memory B cells might be expected to have a relatively low affinity for HBsAg, such that ELISpot had insufficient sensitivity to detect them. Higher affinity B cells detectable by ELISpot are then formed from activation of naïve B cells following the second and third vaccines.

### Distinct features of the vaccine-specific clusters after each vaccine dose

**Fig. 7:**
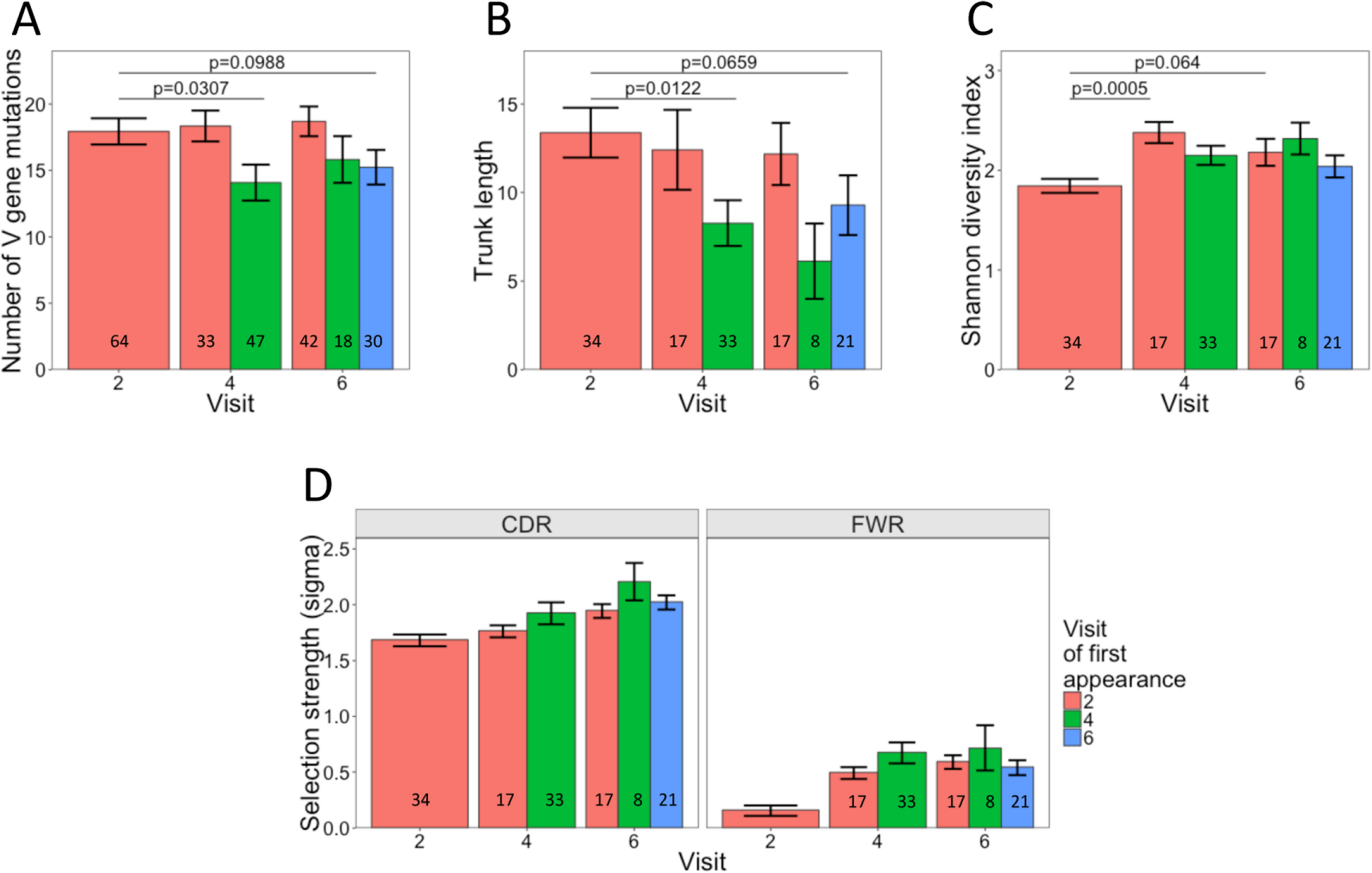
Properties of vaccine-enriched clusters present at each of the three day 7 post-vaccination visits. At each visit, the clusters were split according to the visit when they were first identified. **a** Difference in number of V gene mutations of vaccine-enriched clusters at each visit. **b**, **c** Lineage trees were constructed where these clusters contained at least 25 sequences and the trunk length (**b**) and sequence diversity within the lineage (**c**) calculated for each. Mean values ± SEM are shown and the number of clusters is indicated at the bottom of each bar. Comparisons were performed using a Mann–Whitney U test. See also Additional file 1: Figure S5. **d** Difference in selection strength in the CDR and framework regions (*FWR*) of the clusters identified at different visits. Mean values ± 95 % confidence interval are shown

To investigate the hypothesis that the response to the first vaccine dose resembles cross-reactive memory recall compared with a naïve response after the second two vaccine doses, the properties of the vaccine-specific clusters that were found 7 days after each of the three vaccinations were investigated in more detail. At each visit there are two populations of clusters—those newly generated in response to the vaccine and those that were also present at an earlier visit and are being re-stimulated—so the clusters were split by the visit at which they were first detected in order to reflect this. Considering the mutation level of the clusters, those first found after the second and third vaccine doses were significantly less mutated than those found after the first vaccine dose (Fig. 7a). Those found after the first vaccine dose and also after the later vaccine doses retained a similar level of mutation throughout. Where these clusters contained at least 25 sequences, lineage trees could be constructed from them. The trunk length of the lineages followed a similar pattern to mutation of the clusters. Lineages of clusters first found after the second and third doses had significantly shorter trunk lengths than those found after the first vaccine dose, indicating that the clusters found after the first vaccine dose were derived from more mature precursor cells than those first found after the second and third doses (Fig. 7b). Considering the diversity of sequences within the lineages, this was greater for clusters first found after the second and third vaccine doses compared with those found after the first vaccine dose (Fig. 7c). Furthermore, for the clusters that are present after the first vaccine dose, these became more diverse after the subsequent doses. Analyzing the selection strength of the clusters revealed that the positive selection strength was lower for the clusters found after the first vaccine dose compared with those first appearing after the subsequent vaccine doses (Fig. 7d). So, while the absolute number of mutations is lower in the clusters found after the second and third vaccine doses compared with the first vaccine dose, these mutations are strongly selected for and more likely to result in an amino acid difference.

Taken together, the data presented in Fig. 7 provide evidence that the response to the first vaccine dose is dominated by activation of pre-mutated memory B cells, while less mutated naïve cells are also used in the response to the subsequent doses. It also appears that these pre-mutated memory B cells undergo more diversification than the naïve cells. At visit 6, the timing of the vaccine dose was different in the two vaccine groups, which may have impacted the properties of the lineages, but the study was not powered to detect such effects. It is interesting to note that although not significant, the average trunk length and number of mutations of the lineages is reduced after the third dose in the 0, 1, 2 group compared with the 0, 1, 7 group (Additional file 1: Figure S5).

## Discussion

We used high-throughput sequencing of the human BCR heavy chain repertoire to gain insight into B-cell dynamics following repeated vaccination with an antigen to which the participants had no prior exposure (HBsAg). We show that vaccine-induced changes in the total repertoire following vaccination involve a small minority of sequences, making them challenging to distinguish from background fluctuations. By focusing on the vaccine antigen-specific repertoire, we were able to gain clearer and more detailed insight into the vaccine-induced B-cell dynamics than has previously been possible. We were thus able to demonstrate that a large proportion of the responding sequence clusters are present at baseline in an IgG population (~50 %) and have features which suggest that they are a mature population previously generated in response to a presumably unrelated antigen. The involvement of these clusters in response to HepB vaccine suggests that they also have a degree of cross-reactivity with HBsAg.

We have previously demonstrated that, in the context of a HepB booster vaccine, the features of the global B-cell repertoire change (increase in cluster expansion, increase in mutation, increase in repertoire convergence between participants, and decrease in CDR3 length) [2]. These changes also occur in the current dataset and, as before, are somewhat obscured by background fluctuations in the non-specific repertoire of these individuals that obscure the vaccine-specific effects [10]. Additional noise may also come from the use of two different vaccination schedules. Furthermore, strong hallmarks of repertoire activation were observed in at least two of the participants in this study prior to any vaccination, highlighting the need to focus on the vaccine-specific repertoire rather than the total repertoire. To this extent, we used cell sorting to isolate and sequence vaccine-specific cells and used a strict procedure to identify the vaccine-specific clusters within the total repertoire. We note, however, that despite referring to these clusters as “vaccine-specific”, we cannot be certain of their specificity as we are unable to express and characterize them due to lack of having a paired light chain. This is a limitation that should be removed in future studies as techniques for high-throughput pairing of heavy and light chains emerge [24]. Comparison of our vaccine-specific clusters to random size-matched clusters does, however, reinforce our idea that they are specific to the vaccine as they tend to be more recently activated from germline and undergo increased diversification during the course of the study. It is worth noting that it is necessary to bear in mind some limitations when interpreting lineage tree data. First, sequencing and PCR errors introduce some erroneous sequences, which may be evident in the lineages as large canopies of small closely related nodes (erroneous) arising from the larger central nodes (correct). Such error is evident in the example size-matched lineage (Fig. 4h) and reduces the accuracy at which lineage diversity can be calculated. While methods do exist to try and remove erroneous sequences [25], we did not use them here due to concern that real sequences arising from somatic hypermutation could also be removed (perhaps in a biased manner), thus reducing our sensitivity of detecting true diversification. Instead, we prefer to be cognizant of the error and take this into account during interpretation of the data. Any error should systematically affect all lineages in the same way and so should not affect the conclusions presented here. In fact, if the large canopies of the size-matched lineages are truly erroneous, it would further decrease their diversity in relation to the vaccine-enriched lineages. For future work, molecular barcoding could be used to reduce the impact of such error [26]. Furthermore, it is currently not possible to conduct lineage assignment with complete confidence, so some lineages may contain distinct erroneous nodes which may be members of different lineages [27]. For example, the node in the vaccine-enriched lineage, which is 26 mutations away from the most recent common ancestor of the lineage, could represent such an erroneous node. These erroneous nodes will have little influence on the trunk length measurements but will equally impact all lineages, thus not affecting our broad conclusions (Fig. 4h).

Studying the vaccine-specific repertoire yielded a number of interesting observations. It is striking that prior to vaccination all participants had clusters annotated as vaccine-specific despite never having previously encountered the vaccine antigen. Whilst this may be expected in the naïve repertoire, we sequenced only the class-switched IgG repertoire. This population represents previously activated cells so is not expected to contain cells specific to the antigen. Although finding vaccine-specific sequences at baseline could represent an artifact from incorrect labeling of the vaccine-specific repertoire, we also find sequences matching previously characterized HBsAg-specific antibodies from the literature at baseline, indicating that this is not the case. It seems likely, therefore, that the clusters present at baseline represent B cells that have previously been activated in response to different antigens and that are either polyreactive or happen to have a degree of cross-reactivity with HBsAg. Such a finding is backed up by previous reports which show that polyreactive B cells are a major constituent of the normal human B-cell repertoire [28] and that these may be activated by specific antigens but then require a period of maturation before they develop sufficient specificity to be detected by methods such as ELISpot [29, 30]. Di Niro et al. [29] generated monoclonal antibodies from PCs produced in response to *Salmonella* Typhimurium infection and found that only a tiny fraction produced *Salmonella*-specific antibodies and many appeared to be polyreactive (measured using ELISA and protein microarray). By subsequently characterizing the lineages of the *Salmonella*-specific clones, they showed that initial selection was promiscuous, activating both memory and naïve cells with undetectable affinity for *Salmonella*, and *Salmonella*-specific cells within the lineages could only be detected once there had been a greater degree of affinity maturation. Williams et al. [30] generated monoclonal antibodies from gp41-reactive B cells produced following HIV-1 envelope protein vaccination, finding that many were non-neutralizing and had some polyreactivity, possibly explaining the vaccine failure. Further investigation also revealed ancestors to the gp41-reactive cells were also present prior to vaccination in one individual and were being stimulated in a cross-reactive manner by the vaccine. These results back up our observations that cross-reactive B cells to a novel antigen can be detected in the memory compartment prior to antigen exposure and it will be interesting to uncover the degree to which such a phenomenon actually represents the normal response to any antigen.

Tracking the dynamics of the vaccine-specific repertoire revealed a degree of response after each of the three vaccine doses. After the first vaccine dose, for most participants, the majority of the responding clusters were those that were also present at baseline, highlighting the large extent to which recruitment of these potentially cross-reactive B cells occurs. After subsequent vaccines, there was more recruitment of clusters that are not present at baseline, but some baseline clusters were still recruited. This is indicative, therefore, of recruitment of naïve vaccine-specific cells occurring concomitantly to re-stimulation of the initial cross-reactive cells. It is notable that despite detecting these responses in the sequence data after the first vaccine, no responses were detected by ELISpot. It could be that repertoire sequencing is simply a more sensitive method for detecting the vaccine-specific effects (500,000 B cells used for repertoire sequencing versus 200,000 PBMCs per well for ELISpot) or, alternatively, that, because we suspect most of the B cells activated after the first vaccine were derived from a cross-reactive response, they are likely to have too low affinity for HBsAg for their antibody to be detected by ELISpot. Indeed, this observation is backed up by our findings that, when selectively considering the vaccine-specific clusters present at baseline, their numbers do not correlate with the ELISpot data but, when selectively considering the vaccine-specific clusters not present at baseline, their numbers strongly correlate with the ELISpot data. Plasmablasts that have only recently differentiated and may not yet be secreting large amounts of antibody could also preclude detection by ELISpot [31].

## Conclusions

The data presented here provide insight into B-cell dynamics following repeated antigen stimulus. We show that focusing on the vaccine-specific repertoire is essential to reduce the background noise and that these data yield additional insights beyond those available from conventional techniques such as ELISpot. We uncover a significant role of cross-reactive B cells in the response to HepB vaccine. It will be interesting to investigate whether this also occurs in the response to other vaccines and the degree to which cross-reactive activation affects the level of protection conferred by the vaccine.

## Abbreviations

ASC, antibody-secreting cell; BCR, B-cell receptor; CDR, complementarity determining region; HBsAg, hepatitis B surface antigen; HepB, hepatitis B; PBMC, peripheral blood mononuclear cell; PC, plasma cell

## Additional file

Additional file 1: A PDF file containing Supplementary **Figures S1–S5** and Supplementary **Tables S1–S3**. **Figure S1:** study design and initial sequence processing pipeline. **Figure S2:** HBsAg-specific B-cell response kinetics determined by ELISpot for each individual participant. **Figure S3:** cluster kinetics plots for the total repertoire. **Figure S4:** identification of HBsAg-specific sequences from the literature in our dataset. **Figure S5:** differences in the properties of the visit 6 vaccine-specific clusters in the two vaccine groups. **Table S1:** summary of sequence data obtained from total B-cell samples. **Table S2:** summary of sequence data obtained from HBsAg+ and PC sorted samples. **Table S3:** previously described HBsAg-specific sequences that map to clusters in our dataset. (PDF 1090 kb)
