# Erratum to: B-cell repertoire dynamics after sequential hepatitis B vaccination and evidence for cross-reactive B-cell activation

**DOI:** 10.1186/s13073-016-0337-5

**Published:** 2016-08-03

**Authors:** Jacob D. Galson, Johannes Trück, Elizabeth A. Clutterbuck, Anna Fowler, Vincenzo Cerundolo, Andrew J. Pollard, Gerton Lunter, Dominic F. Kelly

**Affiliations:** 1 Oxford Vaccine Group, Department of Paediatrics, University of Oxford and the NIHR Oxford Biomedical Research Center, Oxford, OX3 7LE UK; 2 Paediatric Immunology, University Children’s Hospital Zürich, Zürich, 8032 Switzerland; 3 Wellcome Trust Centre for Human Genetics, University of Oxford, Oxford, OX3 7BN UK; 4 Medical Research Council Human Immunology Unit, Weatherall Institute of Molecular Medicine, Oxford, OX3 9DS UK

## Erratum

It has come to our attention that there was an omission in the Acknowledgements section in this article [1]. The Acknowledgements section should read:

The authors are grateful to the study participants, to the doctors and nurses at the Oxford Vaccine Group for assisting with sample collection, and to the National Institute for Health Research Clinical Research Network. The authors thank Craig Waugh for help with cell sorting and the High-Throughput Genomics Group at the Wellcome Trust Centre for Human Genetics (subsidized by Wellcome Trust grant reference 090532/Z/09/Z) for the generation of sequencing data. Purified HBsAg was provided by GlaxoSmithKline Biologicals SA, and conjugated to APC by Miltenyi Biotec.
