# Supplementary material for: B-cell repertoire dynamics after sequential hepatitis B vaccination and evidence for cross-reactive B-cell activation
Source: Genome Med. 2016 Jun 16;8:68. doi: 10.1186/s13073-016-0322-z (PMC4910312; doi:10.1186/s13073-016-0322-z)
Supplement: Additional file 1: — A PDF file containing Supplementary Figures S1–S5 and Supplementary Tables S1–S3. Figure S1: study design and initial sequence processing pipeline. Figure S2: HBsAg-specific B-cell response kinetics determined by ELISpot for each individual participant. Figure S3: cluster kinetics plots for the total repertoire. Figure S4: identification of HBsAg-specific sequences from the literature in our dataset. Figure S5: differences in the properties of the visit 6 vaccine-specific clusters in the two vaccine groups. Table S1: summary of sequence data obtained from total B-cell samples. Table S2: summary of sequence data obtained from HBsAg+ and PC sorted samples. Table S3: previously described HBsAg-specific sequences that map to clusters in our dataset. (PDF 1090 kb) [file 13073_2016_322_MOESM1_ESM.pdf]

Supplementary figures and tables

Galson *et al.* B cell repertoire dynamics after sequential Hepatitis B vaccination, and evidence for cross-reactive B cell activation

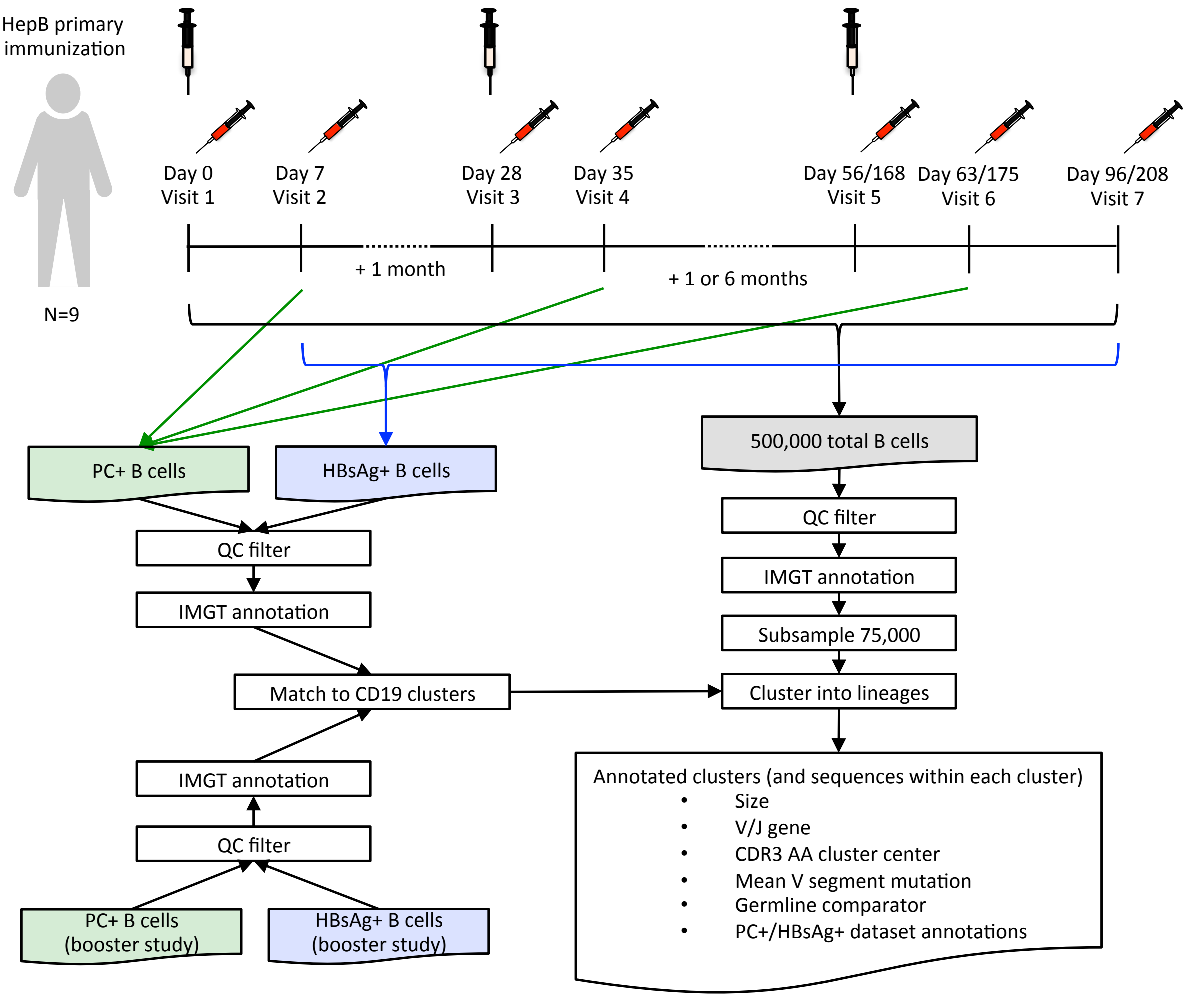

Figure S1. Schematic of study design, and initial sequence processing pipeline. Nine participants were given a three dose primary course of HepB immunization. Five participants were given the conventional schedule (month 0, 1 & 2), and four participants an accelerated schedule (month 0, 1 & 7). Blood was taken on the day of each vaccine, 7 days after each vaccine, and one month after the final vaccine. IgG transcripts from 500,000 total B cells were sequenced at each timepoint. Sequences were submitted to IMGT for annotation, and clustered into clonal lineages. PC's were sorted and sequenced at the day 7 visits, and HBsAg+ B cells were sorted and sequenced on visits 2-7. Sequences from the HBsAg+ and PC's were used to find clusters in the total repertoire that were enriched for specificity towards the vaccine. In addition, sequences from HBsAg+ and PC's from a previous HepB booster vaccine study conducted in our lab [2] were used to find further clusters in the total repertoire that were enriched for specificity towards the vaccine.

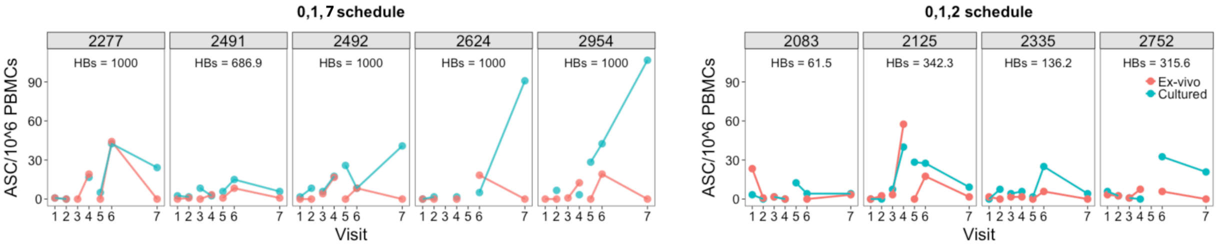

Figure S2. HBsAg-specific B cell response kinetics determined by ELISpot for each individual participant. PBMC's are used ex-vivo to detect PCs, and are cultured to detect memory cells. Note that some points are missing due to either assay failure, or insufficient blood volume collected for the sample. The number at the top of each plot shows the anti-HBsAg antibody concentration at the end of the study for that participant.

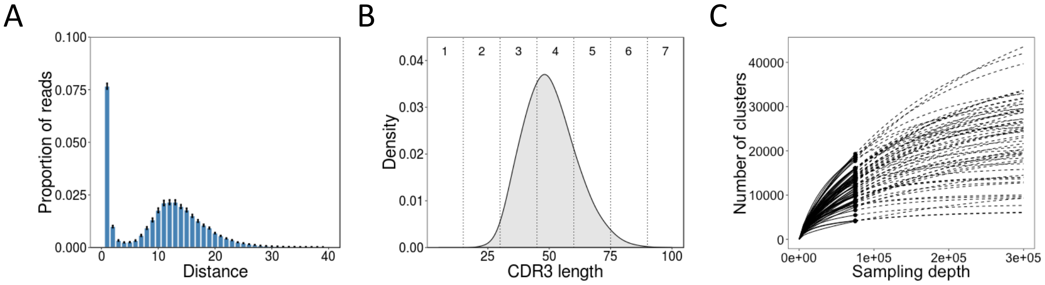

Figure S3. Determining clustering thresholds, and performing rarefaction. (A) Nearest neighbor distribution of CDR3 nucleotide sequences before clustering. For each sample, the nearest neighbor of each sequence was determined by comparing it to every other sequence of the same length in the dataset to find the closest match. The distance is the number of nucleotide differences between the sequence and its nearest neighbor. Distributions were calculated from all samples, and mean values ± SEM plotted. (B) Density plot showing the distribution of CDR3 nucleotide sequences of different lengths in the dataset. The horizontal lines show where the length-based clustering cutoffs are (1 mismatch per 15 nucleotides), and the number between each line is indicative of the maximum number of mismatches that are allowed between sequences of that length to be considered part of the same cluster. (C) Extrapolated rarefaction curves generated from the clustered dataset for each sample. Rarefaction was conducted by subsampling data without replacement at 1,000 sequence increments, and determining the number of clusters represented by these sequences (solid line). Extrapolation (dotted line) was based on Chao's estimator formula, and conducted up to a depth of 300,000 sequences.

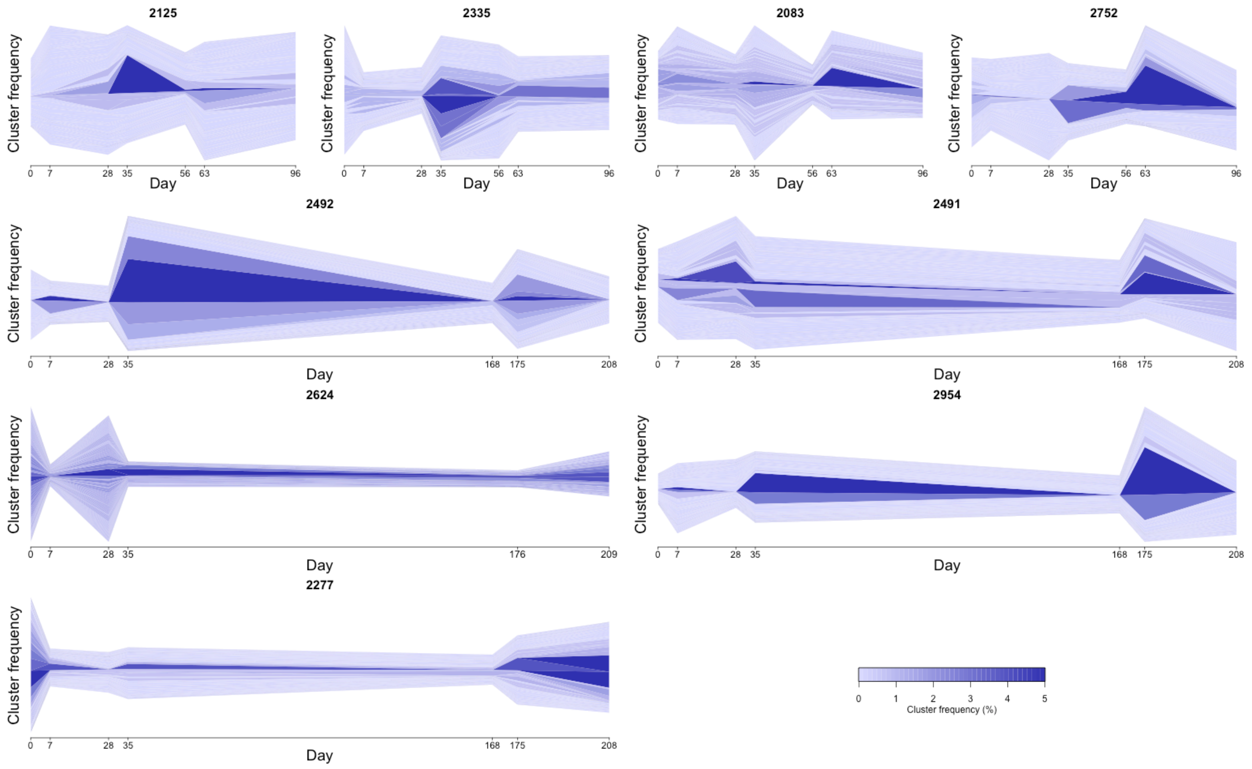

Figure S3. Cluster kinetics plots for the total repertoire. For each participant, the 200 most frequent clusters are found. At each day, the frequencies of these clusters are plotted as a stacked bar chart, centered to the middle of the y axis. Clusters from each day are then joined using a horizontal stream to illustrate how the frequency of the clusters changes over time. The width and darkness of the stream represents the frequency of the cluster at that time. The top four plots are from participants who were given the accelerated vaccine schedule, and the bottom five plots are from participants who were given the conventional vaccine schedule.

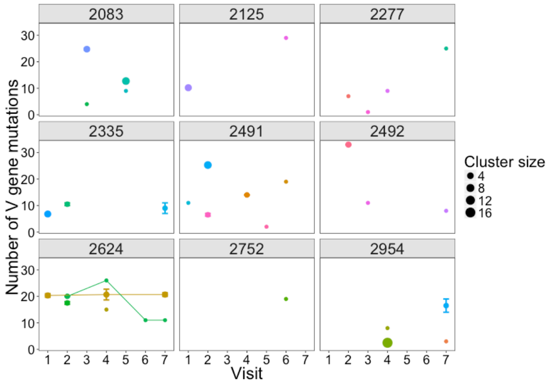

Figure S4. Identification of HBsAg-specific sequences from the literature in our dataset. Previously described sequences were mapped to our dataset to see which clusters they would fall into based on CDR3 AA sequence identity (Table S3). Shown are visits when these clusters are present in each participant. Size of the spot indicates the number of sequences in the cluster at that timepoint, and the position on the y axis indicates the mean mutation of the sequences within the cluster at that timepoint. Where there were multiple sequences in the cluster, error bars showing ± SEM of the mutation were plotted.

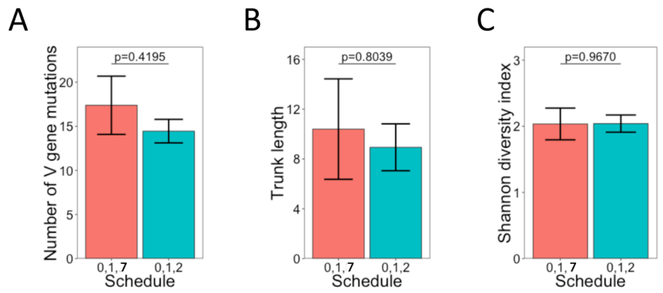

Figure S5. Differences in the properties of the visit 6 vaccine-specific clusters in the two vaccine groups. Differences between the two vaccine groups were investigated for the third vaccine only, which was given at a different time for the two groups. (A) The clusters unique to visit 6 were identified, and the mean number of V gene mutations of these clusters determined. In addition, lineage trees were then constructed for each of these clusters where there were at least 25 sequences, and trunk length (B), and sequence diversity within the lineage (C) calculated. Shown are the mean values ± SEM. Comparisons were performed using the Mann-Whitney U test.

Table S1. Summary of sequence data obtained from total B cell samples.

Participant ID	Visit	Day	Cell number	Raw sequences	Filtered sequences	Subsampled sequences	Clusters
2125	1	0	500000	280250	109983	75000	9749
2125	2	7	500000	248724	91470	75000	6574
2125	3	28	400000	271568	107778	75000	8378
2125	4	35	480000	282535	106228	75000	10900
2125	5	56	440000	240033	88508	75000	15931
2125	6	63	480000	273632	96988	75000	14404
2125	7	96	500000	314920	106679	75000	10009
2335	1	0	500000	326107	123059	75000	5467
2335	2	7	500000	265573	95504	75000	7579
2335	3	28	500000	295070	110753	75000	8885
2335	4	35	500000	292074	109891	75000	8556
2335	5	56	500000	297512	115352	75000	9969
2335	6	63	500000	278008	105298	75000	12329
2335	7	96	500000	292518	100159	75000	11578
2083	1	0	500000	285665	101829	75000	7804
2083	2	7	80000	289428	107976	75000	12502
2083	3	28	500000	316854	110819	75000	14668
2083	4	35	360000	287595	106748	75000	7593
2083	5	56	500000	290731	96961	75000	18166
2083	6	63	240000	305533	110727	75000	18754
2083	7	96	440000	241392	75872	75000	18763
2752	1	0	500000	329479	126338	75000	8302
2752	2	7	500000	341075	129083	75000	10572
2752	3	28	500000	307994	110134	75000	9779
2752	4	35	500000	341418	124074	75000	12132
2752	5	56	500000	332007	125062	75000	16027
2752	6	63	500000	287170	105604	75000	14988
2752	7	96	500000	345096	122024	75000	11520
2492	1	0	200000	325226	130221	75000	9588
2492	2	7	280000	342157	122949	75000	13864
2492	3	28	360000	348220	114600	75000	14119
2492	4	35	500000	337566	134155	75000	4081
2492	5	168	500000	322941	117357	75000	16067
2492	6	175	500000	332830	123540	75000	8745
2492	7	208	500000	302128	102733	75000	11150

2491	1	0	500000	326473	120009	75000	16110
2491	2	7	500000	331065	117952	75000	16074
2491	3	28	500000	332481	119947	75000	14070
2491	4	35	500000	312307	108437	75000	13497
2491	5	168	500000	352448	125808	75000	17688
2491	6	175	500000	338750	122524	75000	19304
2491	7	208	500000	355851	124847	75000	8767
2624	1	0	120000	277691	106745	75000	4245
2624	2	7	500000	328034	129485	75000	12250
2624	3	28	120000	301307	104030	75000	4192
2624	4	35	500000	316346	109994	75000	13632
2624	6	175	500000	249755	81514	75000	15646
2624	7	208	120000	297572	99247	75000	13037
2954	1	0	500000	321396	121817	75000	18159
2954	2	7	500000	317080	115104	75000	13229
2954	3	28	500000	298952	103096	75000	13504
2954	4	35	500000	314153	114472	75000	14418
2954	5	168	500000	293970	103834	75000	18728
2954	6	175	500000	324087	119884	75000	14816
2954	7	208	500000	318612	102392	75000	16058
2277	1	0	500000	342026	136140	75000	7249
2277	2	7	500000	339324	107606	75000	13510
2277	3	28	500000	327746	120260	75000	11884
2277	4	35	500000	269894	92774	75000	10393
2277	5	168	500000	336158	118025	75000	13776
2277	6	175	500000	293158	100787	75000	13210
2277	7	208	500000	315752	101956	75000	10253

Table S2. Summary of sequence data obtained from HBsAg+ and PC sorted samples.

Participant ID	Visit	Day	Cell subset	Cell number	Raw sequences	Unique filtered sequences
2125	2	7	HBsAg+	1628	59493	778
2125	3	28	HBsAg+	1963	333954	985
2125	4	35	HBsAg+	3569	38023	4582
2125	5	56	HBsAg+	534	304276	590
2125	6	63	HBsAg+	2239	28173	1736
2125	7	96	HBsAg+	772	343337	1146
2335	2	7	HBsAg+	4956	300768	4558
2335	3	28	HBsAg+	1963	58977	6077
2335	4	35	HBsAg+	10000	269371	6702
2335	5	56	HBsAg+	1363	59109	1161
2335	6	63	HBsAg+	3056	333485	4130
2335	7	96	HBsAg+	2866	53465	1371
2083	2	7	HBsAg+	1840	11488	2435
2083	3	28	HBsAg+	2914	287257	2232
2083	4	35	HBsAg+	3670	70153	6435
2083	5	56	HBsAg+	1365	345063	1131
2083	6	63	HBsAg+	1285	63585	1634
2083	7	96	HBsAg+	695	351756	343
2752	2	7	HBsAg+	1689	317723	1265
2752	3	28	HBsAg+	2303	56544	981
2752	4	35	HBsAg+	12000	344405	8401
2752	5	56	HBsAg+	4260	23743	6770
2752	6	63	HBsAg+	3201	342352	7478
2752	7	96	HBsAg+	2848	47866	2114
2492	2	7	HBsAg+	4337	55272	8049
2492	3	28	HBsAg+	5833	440076	8001
2492	4	35	HBsAg+	4521	29642	6210
2492	5	168	HBsAg+	2339	292343	149
2492	6	175	HBsAg+	2545	67994	5504
2492	7	208	HBsAg+	3174	374457	7058
2491	2	7	HBsAg+	10000	291482	8239
2491	3	28	HBsAg+	3139	70670	6469
2491	4	35	HBsAg+	7045	302927	9152
2491	5	168	HBsAg+	8812	48948	101
2491	6	175	HBsAg+	6344	405350	2565
2491	7	208	HBsAg+	6687	77866	7994
2624	2	7	HBsAg+	8129	48770	7098

2624	3	28	HBsAg+	2677	516659	2365
2624	4	35	HBsAg+	7837	14744	6419
2624	6	175	HBsAg+	4699	102802	6201
2624	7	208	HBsAg+	1871	14869	1633
2954	2	7	HBsAg+	9041	48523	10978
2954	3	28	HBsAg+	5673	363442	10372
2954	4	35	HBsAg+	6097	8155	9985
2954	5	168	HBsAg+	4273	305422	90
2954	6	175	HBsAg+	4536	3570	11975
2954	7	208	HBsAg+	7869	203874	8238
2277	2	7	HBsAg+	3912	323748	6911
2277	3	28	HBsAg+	5136	278108	8402
2277	4	35	HBsAg+	8563	322579	8538
2277	5	168	HBsAg+	10000	172336	130
2277	6	175	HBsAg+	7104	298652	5190
2277	7	208	HBsAg+	5312	290192	7390
2125	2	7	PC	912	94768	4167
2125	4	35	PC	1350	111820	5819
2125	6	63	PC	272	87461	2763
2335	2	7	PC	1594	102694	5526
2335	4	35	PC	5603	120645	6607
2335	6	63	PC	364	111386	4116
2083	2	7	PC	130	103870	1490
2083	4	35	PC	350	93350	2225
2083	6	63	PC	143	123015	896
2752	2	7	PC	613	95954	3942
2752	4	35	PC	1814	114910	4994
2752	6	63	PC	420	98120	3473
2492	2	7	PC	626	118753	3009
2492	4	35	PC	2187	114854	3207
2492	6	175	PC	483	130166	4526
2491	2	7	PC	427	114667	3462
2491	4	35	PC	479	116859	4082
2491	6	175	PC	593	104336	13365
2624	2	7	PC	551	126379	4407
2624	4	35	PC	839	117360	5673
2624	6	175	PC	690	107392	5016
2954	2	7	PC	977	126511	6380
2954	4	35	PC	1283	118926	5984
2954	6	175	PC	702	112572	4754
2277	2	7	PC	169	75811	1997

2277	4	35	PC	262	118938	3843
2277	6	175	PC	897	116250	4484

Table S3. Previously described HBsAg-specific sequences that map to clusters in our dataset.

CDR3 AA sequence	Reference
CTAGFDYW	IMGT/LIGM-DB
CARDLELW	IMGT/LIGM-DB
CARGFHYW	Becker et al. PLoS ONE. 2010
CARGLSGFDYW	IMGT/LIGM-DB
CTRKSSSSDYW	Becker et al. PLoS ONE. 2010
CARGGFGEFDYW	IMGT/LIGM-DB
CARGTVGYWFDPW	IMGT/LIGM-DB
CARDAIAVASDYW	Tajiri et al. Antiviral Research. 2010
CTRTYSSSWYFDYW	Becker et al. PLoS ONE. 2010
CAKDIASWYYYGMDVW	Becker et al. PLoS ONE. 2010
CAKAIASWYYYGMDVW	Tajiri et al. Antiviral Research. 2010
CAKDISSWYYYGMDVW	Jin et al. Nature medicine 2009
